# Beyond Lip Service: A Position Paper to Truly Stimulate Shared Decision‐Making

**DOI:** 10.1111/jan.70053

**Published:** 2025-07-22

**Authors:** Maureen Thodé, Jeroen Dikken, Prabath W. B. Nanayakkara

**Affiliations:** ^1^ Department of Internal Medicine, Section General Internal Medicine Amsterdam UMC Location Vrije Universiteit Amsterdam Amsterdam the Netherlands; ^2^ Research Group of Urban Ageing, Faculty of Social Work & Education The Hague University of Applied Sciences The Hague the Netherlands; ^3^ Faculty of Health, Nutrition & Sport The Hague University of Applied Sciences The Hague the Netherlands

**Keywords:** nurse involvement, nursing, patient empowerment, patient‐centred care, shared decision‐making, treatment decision‐making

## Abstract

**Aim:**

To discuss how shared decision‐making (SDM) is currently practised in hospitals, to highlight the essential—yet often underacknowledged—contribution of nurses to inclusive SDM in life‐prolonging treatment decisions, and to propose a five‐step implementation plan to strengthen the role of patients in the SDM process.

**Design:**

A position paper on current SDM practices.

**Methods:**

To take a position, we drew on knowledge gained from six empirical studies conducted by our research group and evaluated these findings in light of the most recent literature.

**Results:**

A five‐step implementation plan to stimulate SDM: (1) Clarify roles, (2) Organisational alignment, (3) Comprehensive training, (4) Tailored implementation plans, and (5) Sustainable integration.

**Conclusion:**

The plan is ambitious, yet it offers a clear and actionable path forward for healthcare organisations and professionals. It provides a concrete opportunity for collaboration to embed SDM in daily clinical practice. Ultimately, our shared objective is to achieve optimal patient outcomes—an aim that unites all stakeholders.

**Implications for the Profession and/or Patient Care:**

Integrating nurses into SDM processes will enhance the quality of support for treatment decision‐making. However, to realise truly inclusive, high‐quality, patient‐centred care, coordinated action at multiple organisational levels is essential.

**Impact:**

The proposed plan is not only relevant to treatment decisions at the end of life in hospital settings, but also presents broader opportunities to advance SDM across healthcare sectors. It offers nurses a clearly defined and meaningful role in SDM and provides a practical blueprint for implementation at all levels of the organisation—transforming long‐standing ambitions into tangible practice.

## 1 Introduction

In contemporary hospital healthcare, truly placing patients at the centre of life‐prolonging treatment decisions remains a persistent challenge, and shared decision‐making (SDM) often falls short in day‐to‐day clinical practice (Fisher et al. 2018; Noteboom, Vervoort, et al. 2021; Kuijpers et al. 2022; Staveley and Sullivan 2015).

For example, many patients in their last phase of life do not realise that they have choices among various treatment options, each with distinct advantages and disadvantages (Brom et al. 2014; Shrestha et al. 2019). Instead, physicians often present a single life‐prolonging treatment option they consider most suitable, typically during brief consultations intended to solicit the patient's values, preferences, and cultural beliefs (Staveley and Sullivan 2015). Although unintended, this approach often steers the patient towards physicians preferred treatment choices (Pieterse et al. 2022).

This practice reflects the current interpretation of SDM by healthcare professionals, wherein decision‐making is streamlined to align within the immediate clinical encounter. However, the SDM model is designed to unfold over multiple conversations, spanning three or four distinct steps in order to truly place the patients at the centre (Elwyn et al. 2017; Stiggelbout et al. 2015). This paper applies the widely adopted four‐step model by Stiggelbout et al., which structures SDM as: recognising a decision is needed, presenting options, supporting preference formation, and making or deferring a joint decision with planned follow‐up (Stiggelbout et al. 2015; Elwyn et al. 2017). This ultimately should result in a treatment decision that authentically reflects the patient's preferences, cultural convictions, and pursuit of the highest quality of life and satisfaction with the treatment decision at that moment in their disease trajectory (Kane et al. 2014; Kunneman et al. 2016; Stiggelbout et al. 2012). In reality, however, this process is frequently condensed into a single step, which undermines the broader principles of SDM (Kuijpers et al. 2022; Staveley and Sullivan 2015).

This raises a pivotal question: how does this constrained application of the SDM model genuinely serve the patient's best interests and uphold the fundamental principles we aspire to honour in daily hospital practice?

Meaningful participation in SDM often requires support, as healthcare discussions frequently extend beyond patients' everyday experiences (Keij et al. 2024; Noteboom, Vervoort, et al. 2021). This support is particularly important for individuals with limited (health) literacy or expatriates, who may struggle with unfamiliar systems, communication norms, and culturally defined roles (Dobler et al. 2017; Muscat et al. 2019; Rademakers 2014). Cultural or ethnic differences between patients and providers may further complicate the process, as clinicians' preferences can be shaped by their own cultural context, influencing decision‐making dynamics.

Most research on cultural influences in SDM has centred on the United States, primarily comparing White and African American populations (Peek et al. 2010), leaving other contexts underexplored. Culture affects decision‐making through varying perceptions of risk, trust, family involvement, and role preferences (Mead et al. 2013). In some groups, such as those of Asian and Caribbean descent (as supported by our experience), paternalism and collective decision‐making are often valued, underscoring the need for culturally aligned care (Pun et al. 2023). Applying Western SDM models without adaptation can result in communication gaps and unmet expectations.

Structural barriers, such as complex medical language, may also discourage patient engagement. Even when invited to participate, patients may defer to the physician's judgement—asking, “Doctor, what do you think?”—leading to decisions made without full understanding or personal alignment (Noteboom, May, et al. 2021; Kane et al. 2014). This compromises SDM and may reduce satisfaction and quality of life, particularly in end‐of‐life care, where a culturally responsive approach is crucial.

Some patients actively seek information to better understand their illness and participate in decisions (Tan and Goonawardene 2017). A small group, often with higher education and strong communication skills, successfully evaluate sources and contribute meaningfully (Epstein et al. 2017; Tan and Goonawardene 2017). Others struggle to assess the credibility of information, relying on healthcare providers to help clarify values and guide choices (Elwyn et al. 2009). This becomes particularly critical in end‐of‐life decisions, where treatments such as chemotherapy or artificial nutrition involve complex trade‐offs. These decisions must be grounded in the patient's values, preferences, and cultural beliefs to balance survival with quality of life (Brom et al. 2014; Shrestha et al. 2019).

When navigating these complex decisions, SDM could serve as a crucial framework, merging medical expertise with the patient's unique individual values and priorities (Elwyn et al. 2017; Stiggelbout et al. 2015). To realise genuine patient‐centred care, clinicians must present all available options rather than defaulting to a single recommended treatment (Stiggelbout et al. 2015). However, the manner in which options are conveyed is equally important. Complex medical language may deter engagement, prompting patients to defer to the physician without seeking clarification. This highlights the pressing question of who is responsible for supporting patients throughout the decision‐making process to ensure that choices genuinely reflect their preferences and needs.

### 1.1 Who? Key stakeholders in the decision‐making process

Prior to the 1980s, healthcare decision‐making was largely paternalistic, with physicians exercising primary authority and prioritising diagnosis and treatment over patients' subjective experiences (Charles et al. 1999; De Haes 2006). A gradual shift towards patient‐centred care followed, driven by expanded treatment options, growing patient assertiveness, and evolving societal and medical values (Butalid et al. 2014; Gattellari et al. 2001). This shift reflected increasing recognition of the need to address illness holistically, incorporating patients' individual needs into clinical decision‐making.

Since the 1980s, fostering patient‐centred care has centred on establishing a balanced distribution of roles among stakeholders, ensuring each participant's contributions serve the patient's best interests. Murray et al. (2004) already stated that collaboration among healthcare providers, patients, their close relatives, and nurses brings unique strengths to the decision‐making process, aligning treatment decisions with the patient's values, preferences, ethnic, and cultural background.

In my opinion it's about sharing. And that applies to me too. You could say I'm the expert on the subject of me, and the doctor is the expert when it comes to the treatment and I reckon the two need to come together. (Incurable patient with glioblastoma multiforme, citated from Brom et al. 2014)

These findings were reinforced by Murray (2010), underscoring the enduring importance of such partnerships. Subsequent studies by Brom et al. (2014) and Stiggelbout et al. (2012) further emphasised the importance of recognising patients as active participants in decision‐making, particularly during the final phase of life.

In these cases, guidance and coaching from healthcare professionals are crucial for helping patients navigate complex treatment choices, empowering them to take an informed and active role in their care.

**FIGURE 1:**
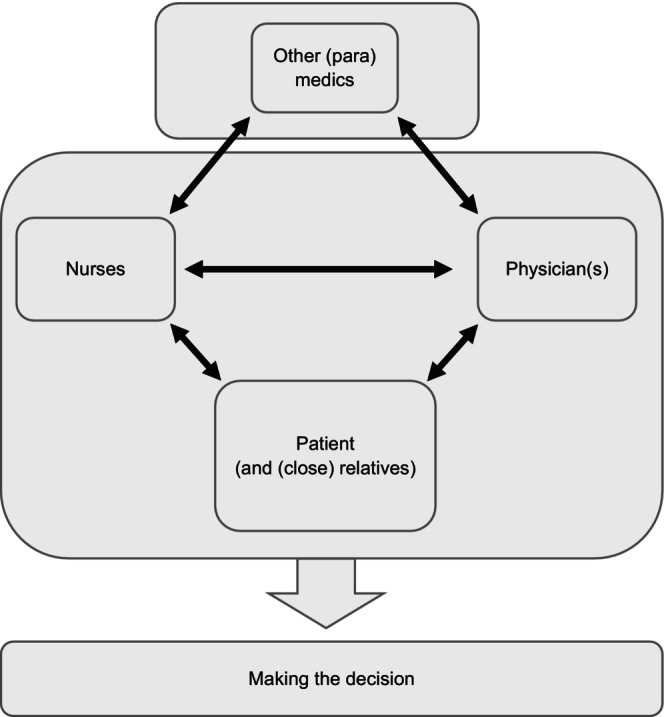
Ideal shared decision‐making (SDM) structure.

The patient is central to the decision‐making process, and both the nurse and the physician should work alongside them to facilitate this (Figure 1). For patients, assuming an active role in decision‐making can be challenging for various reasons.

Yes, the doctor knows what's best for me. I can't judge that from a medical point of view. That's why I think the doctor should decide. […] Well, the actual decision is the doctor's, because medically speaking she… I mean, I don't know anything about the subject. I haven't had any kind of medical training, so I don't know any of that kind of thing, so I just go by what the doctor says. (Incurable patient with cancer, citated from Brom et al. 2014)

Thodé et al. (2020), in a systematic review, showed that providing patients, their close relatives, and healthcare professionals with appropriate tools and guidance fosters more active involvement in treatment decisions. Such support enhances both patient engagement and the overall care experience (Kunneman et al. 2016). Close relatives often offer emotional support and help communicate the patient's preferences, making them valuable contributors to the decision‐making process (Pun et al. 2023). Many patients involve partners, friends, or family members to ensure more informed choices, though some prefer to decide independently. Accordingly, the term close relatives is used in parentheses in Figure 1 to reflect these individual differences. Their involvement, while often beneficial, may also lead to conflicting views with the patient or care team.

Physicians remain central to healthcare, utilising their medical expertise to fulfil essential responsibilities such as diagnosing conditions, initiating treatment discussions, sharing critical information, and guiding patients through decision‐making processes (Arends et al. 2023). They also hold ultimate accountability for decisions concerning life‐prolonging interventions (Arends, Thodé, et al. 2022; Arends et al. 2023).

Complementing their role, nurses provide continuous support throughout the decision‐making process, ensuring that patients are well‐informed and feel supported at every stage.

I prefer to explain the options, after which the nurse gets the opportunity to go through those options once more. Because the patient might have questions about those options. To discuss those options with the patient and only then to decide, so the decision comes after that. So the nurse clarifies the options. (Otolaryngologist, citated from Arends et al. 2023).

Each stakeholder contributes essential expertise to support the patient in selecting the most appropriate treatment. Although the patient's voice typically holds significant weight, other considerations may occasionally take precedence. In rare cases, the physician may assume greater responsibility and make a decisive choice. For instance, when a palliative patient requests curative chemotherapy against medical advice, the physician may decline based on a life expectancy of under 4 months and a treatment cost of €250,000—highlighting broader societal implications.

Nurses complement the physician, the patient (and their close relatives) (Bos‐van den Hoek et al. 2021), and the broader paramedical team involved in care. Serving as a vital bridge, they help ensure that treatment decisions align with the patient's ethnic and cultural background and their core values (Dees et al. 2018).

They [nurses] sometimes speak separately to the family caregiver. [.] That can give us different information to when you speak to them both [the patient and the family caregiver] in the consulting room together. That information sometimes gives us a much more complete picture, or a more complex one, and sometimes it's very much in line with how you already saw the situation. So the one really complements the other. (Geriatrician, citated from Arends et al. 2023)

What's often striking is that they have a better view of what isn't going well with a patient and they act as a brake on you. I know it was the case with myself in the past—and this is connected indirectly—that as a doctor you're sometimes too busy curing the patient and then you get feedback from the nurse saying, things are not going at all well with that patient; we don't have nearly as much confidence as you do. So that feedback is important. (Orthopedic surgeon, citated from Arends et al. 2023)

Although often underacknowledged and limited in their involvement, nurses should actively participate in the team by contributing their expertise and fulfilling their defined role within the SDM process. Additionally, while other disciplines do not directly contribute to the decision‐making process, they support the physician and nurse by providing relevant information, such as on nutrition or exercise. These disciplines act as a second team, offering crucial insights to help the physician, nurse, and patient make the most appropriate treatment decision. Together, patients, their close relatives, the nurse, and the physician should form a cohesive team, fostering effective and patient‐centred healthcare decision‐making, with the patient always at the centre.

It's not entirely true to say I take the final decision–you do it together. The two sides need to come to a kind of agreement, an intuitive one too. And then you subject yourself to it as a patient. Because look, of course you don't have all the know‐how, most patients don't know anything at all. […] It's very difficult for most patients to get a good impression of what the treatment involves, what their clinical picture is, so of course that makes it quite a difficult decision. Sure, most people can still decide whether or not to subject themselves to it, of course, but as far as I'm concerned it's a case of taking a joint decision to go down that route, but definitely after discussing things together and after I've had a think about it. (Patient with glioblastoma multiforme, citated from Brom et al. 2014)

**FIGURE 2:**
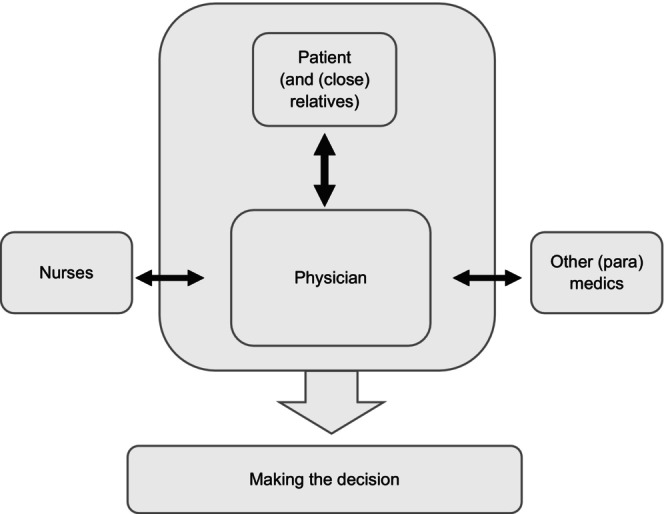
Daily practice shared decision‐making (SDM) structure.

In contrast, daily hospital practice often follows a different structure. The literature suggests that, despite advocating for SDM, daily hospital practice often fails to achieve this ideal. The decision‐making process typically occurs between the physician and the patient, with a close relative occasionally involved (Figure 2). The physician often makes the final decision regarding life‐prolonging treatment, and the patient is usually only in direct relation to the physician.

The physician may also engage with the nurse, though this is not standard practice, and other disciplines are involved when necessary, though not regularly.

Physicians frequently neglect to discuss all treatment options, including the option of forgoing treatment (Kuijpers et al. 2022), while patients tend to focus more on survival than on participating in the SDM process (Noteboom, Vervoort, et al. 2021). Additionally, a paternalistic approach by physicians still limits patients' perception of having a genuine choice in treatment decisions (Noteboom, Vervoort, et al. 2021).

But ultimately I have the final word; I decide whether someone dies. (Nephrologist, citated from Arends et al. 2023)

However, the issue extends beyond these individual shortcomings, as SDM has not yet reached its intended standard. Physicians often fail to engage patients in exploring their values and preferences regarding treatment (Staveley and Sullivan 2015) and struggle to present information in an understandable and unbiased manner (Fisher et al. 2018; Kuijpers et al. 2022). Furthermore, physicians inadequately communicate the consequences of decisions (Kuijpers et al. 2022), provide insufficient time for patient deliberation (Fisher et al. 2018), and tend to overlook individualised patient needs (Kuijpers et al. 2022). Consequently, this approach does not constitute true SDM.

Thus, following the SDM principle, all parties involved in the decision‐making process should have an (equal) voice, though one stakeholder may, at times, have greater influence than others. Healthcare and science have consistently emphasised patient‐centred care, where services are designed to prioritise the patient (Stiggelbout et al. 2012, 2015). For example, if a physician makes a treatment decision that the patient disagrees with and the patient desires an alternative, should the decision process remain equal, as the literature asserts? While the literature advocates for SDM, it is clear that when a doctor states the patient ultimately decides whether to proceed with treatment, this is not truly an equal process. We listen to all perspectives, with nurses providing support, but the final choice must remain the patient's.

This raises a key question: should the final decision in SDM be made collectively by all stakeholders, or should it prioritise in all cases the patient's choice, given that they must undergo the treatment and live with its outcomes?

This dilemma becomes particularly acute during the limited timeframe of the palliative phase. It raises the question: when should physicians deviate from the ideal of full patient autonomy? For example, should treatment options C, D, and E be presented even if they are not preferred by the physician? While patients may not choose all options, offering a complete overview and guiding them through the elimination of less suitable alternatives fosters informed decision‐making. This strengthens patient commitment and provides a basis for accountability. If a patient selects chemotherapy that requires lifestyle adjustments, such as regular exercise, but fails to comply, the physician may refer back to the agreed plan. In this framework, nurses play a vital role by monitoring progress, facilitating dialogue, and offering ongoing support.

Together with physicians, patients, and close relatives, nurses are integral to the SDM process regarding life‐prolonging treatment. Their involvement is widely recognised in both practice and research as essential to supporting patients in making critical decisions about life‐threatening illnesses (Arends, Thodé, et al. 2022; Bos‐van den Hoek et al. 2021). Their contribution can significantly influence patient outcomes.

### 1.2 Nurses potential role in SDM

First of all, nurses are the profession being the most well positioned to facilitate SDM, especially in life‐extending treatment decisions, due to their close and informal interactions with patients, which often reveal insights about patient preferences and wishes that may not emerge in brief, physician‐led consultations (Epstein et al. 2017; Tariman et al. 2016). Therefore, nurses can act as a bridge between patients and physicians, helping to align treatment choices with patients' ethnic and cultural backgrounds and core values (Dees et al. 2018; Tariman and Szubski 2015; Tariman et al. 2016). Including nurses in SDM will lead to decisions that more accurately reflect what matters most to patients in their remaining time.

I mainly outline what patients find important in their lives, what they expect from the treatment, what they know about the diagnosis. (…). And then the physicians often continue about what to expect from the treatment in this situation.

(Clinical nurse specialist, citated from Bos‐van den Hoek et al. 2021)

Research by Bos‐van den Hoek et al. (2021) explored hospital nurses' perspectives on their involvement in decision‐making for life‐prolonging treatments, identifying key conditions for their engagement. Nurses perceive their influence on treatment decisions as varied, with distinct opportunities to contribute at different stages of the process. They take on three primary roles: assessing decision quality, complementing SDM, and facilitating its implementation.

Nurses guide patients through the decision‐making journey by participating in discussions, simplifying complex information, and helping patients identify their priorities (Arends, Thodé, et al. 2022; Bos‐van den Hoek et al. 2021).

So I think there is definitely a role for the nurse there, to slow things down and give someone space and the opportunity to reflect and think about whether they still want things. (Neurologist, citated from Arends et al. 2023)

They also assess whether these priorities align with the life‐prolonging treatment options being considered (Bos‐van den Hoek et al. 2021). Beyond supporting informed decisions, nurses play an active role post‐diagnosis by reviewing treatment options with patients and their families in a calm and accessible manner (Bos‐van den Hoek et al. 2021).

Then I take the patient aside, after the conversation with the neurologist. Just to hear them repeat what they think they've heard – what they think about it. So I'm really helping them process that discussion, briefly summarizing what the options are. (Clinical registered nurse, citated from Bos‐van den Hoek et al. 2021)

While not replacing the physician, nurses facilitate communication between patients, families, and medical teams (Bos‐van den Hoek et al. 2021; Dees et al. 2018; Tariman and Szubski 2015; Tariman et al. 2016). By developing close relationships, nurses gain insights into patients' daily lives, linking these with physicians' clinical knowledge to support genuine SDM (Arends, Thodé, et al. 2022; Bos‐van den Hoek et al. 2021; Tariman and Szubski 2015).

### 1.3 Why nurses struggle in SDM roles

Considerable evidence underscores the critical role of nurses in supporting patients and their close relatives in treatment decision‐making from multiple perspectives (Arends, Thodé, et al. 2022; Arends et al. 2023; Bos‐van den Hoek et al. 2021). However, nurses encounter significant practical, organisational, and knowledge‐related barriers that hinder their ability to fulfil this role effectively. Despite their willingness to contribute meaningfully to SDM, they are frequently excluded by physicians. This exclusion limits their participation in multidisciplinary discussions and their capacity to support patients during the decision‐making process (Arends, Thodé, et al. 2022; Arends et al. 2023).

Time constraints pose a major challenge, with few nurses reporting adequate opportunities to discuss treatment options with patients. Moreover, their role in SDM is often poorly defined, leading to uncertainty and a lack of confidence. Many nurses identify insufficient training in palliative care, communication, and medical treatment options as a significant barrier to providing effective patient support. Organisational obstacles, such as inconsistent information transfer and the insufficient recognition of nurses' contributions, further diminish their involvement in SDM (Bos‐van den Hoek et al. 2021).

Research by Arends, Steenbergen, et al. (2022) highlights the moral distress nurses experience when excluded from decision‐making, particularly when they are required to implement decisions that conflict with the patient's values or may lead to adverse outcomes.

The moment the treatment became futile, I told the physician that I had difficulties with administering fluids intravenously and that I was quitting performing that treatment [for this patient]. (Clinical registered nurse, citated from Arends, Steenbergen, et al. 2022)

This exclusion often fosters feelings of powerlessness. For instance, nurses may perceive treatments as futile or observe patient suffering without being able to intervene meaningfully. These challenges are compounded by a misalignment of priorities: nurses frequently prioritise comfort and quality of life, whereas physicians may focus on life extension. Unrealistic expectations set by physicians for patients and their families exacerbate frustration and further complicate care delivery.

To address these systemic issues, it is imperative to formalise the role of nurses in SDM and provide them with the necessary training and tools to support patients effectively (Arends, Thodé, et al. 2022; Thodé et al. 2020). Their persistent exclusion and struggles across multiple levels underscore the urgency for systemic transformation.

## 2 The plan for true SDM

To effectively participate in patient‐centred decision‐making and support patients, relatives, and physicians, nurses require clearly defined roles for all stakeholders involved in the decision‐making process. This must be accompanied by targeted skills development, robust organisational support at all levels, and a sustainable implementation strategy.

The plan for true SDM consists of five steps:

1. Clarify roles: Clearly define the roles and responsibilities of all parties involved in SDM, ensuring that nurses, physicians, patients, and relatives understand their (expected) contributions to the process.
2. Organisational alignment: Restructure organisations to prioritise the patient's voice. Policies, workflows, and protocols must be reoriented to ensure that patient needs and preferences remain central at all times. Achieving this requires a cultural shift to create an environment where such changes can be effectively embedded in practice.
3. Comprehensive training: Provide extensive and continuous training for all stakeholders, including the current workforce and future healthcare professionals. Training must be reinforced across all levels to ensure the skills required for SDM are consistently developed and sustained.
4. Tailored implementation plans: Develop implementation strategies that are tailored to the specific needs and contexts of each organisation. These plans must be practical and adaptable to ensure successful integration into everyday practice.
5. Sustainable integration: Establish a long‐term framework for embedding SDM within the healthcare system, ensuring ongoing evaluation and adaptation to maintain its effectiveness over time.

### 2.1 Step 1: Defining roles with a focus on nurses' contributions

Clear role definitions are crucial for effective SDM, ensuring that all stakeholders—including physicians, patients, relatives, and other healthcare professionals—understand their responsibilities in the process. Nurses play a pivotal role, requiring sufficient time, structured handovers, and comprehensive training to fulfil their duties effectively (Arends, Thodé, et al. 2022). Their contributions include assessing decision quality, actively participating in SDM, and supporting its implementation, particularly in decisions about life‐prolonging treatments (Bos‐van den Hoek et al. 2021).

By facilitating well‐informed, high‐quality decisions aligned with patient values and preferences, nurses significantly enhance the SDM process and assist physicians in achieving patient‐centred outcomes. However, while some nurses are fully engaged in these roles, others face barriers such as insufficient education and resources, which limit their capacity to guide patients without causing confusion (Bos‐van den Hoek et al. 2021).

Organisational support is therefore essential to enable nurses to engage fully in SDM. Now that the roles are clearly defined and a vision for effective SDM is established, organisations must align their structures and policies to support this approach, ensuring that nurses can contribute meaningfully to decision‐making processes.

### 2.2 Step 2: Organisational support

To implement the vision outlined above, organisations must prioritise a patient‐centred approach that places the patient's voice at the forefront, rather than defaulting to the perspectives of healthcare professionals. This requires presenting all available treatment options, not just those deemed most appropriate by physicians. Embedding this perspective into practice necessitates formalisation within organisational guidelines, protocols, and policies. Once these foundational changes are established, healthcare professionals can be held accountable for adhering to the principles of patient‐centred care.

Establishing a supportive network is also essential to encourage and sustain behaviours aligned with this approach (Kajamaa and Hurmelinna‐Laukkanen 2022). Significant organisational restructuring is required to foster such a culture. Policies should mandate that clinicians present and discuss all treatment options with patients to ensure informed decision‐making (Kajamaa and Hurmelinna‐Laukkanen 2022; Waddell et al. 2021). Furthermore, organisational support, including dedicated time and a team‐based approach, is critical to empowering nurses to actively engage in SDM.

However, time constraints remain a significant barrier, limiting nurses' ability to participate in patient discussions and manage care transitions effectively (Dobler et al. 2019; Joseph‐Williams et al. 2017). For example, only 22% of nurses report having sufficient time to discuss life‐prolonging treatments with patients, highlighting the urgent need for improved time management strategies within healthcare teams (Arends, Thodé, et al. 2022).

To integrate SDM into routine workflows, organisations must support the process with structured tools and foster a collaborative environment (Nibbelink and Brewer 2018; Scholl et al. 2018). Balancing workloads is also critical, as meaningful patient engagement requires adequate time (Arends, Thodé, et al. 2022). In addition to formal training, role modelling and experiential learning can further enhance nurses' competence in SDM. Cultivating a patient‐centred care culture encourages team collaboration, enabling nurses to prioritise patient needs effectively.

As we reflect on current practices and scientific insights, it is clear that true patient‐centred decision‐making hinges on recognising the indispensable role of nurses in supporting patients through life‐extending or non‐life‐extending decisions. By embedding these principles into organisational culture and practice, healthcare teams can truly centre the patient in decision‐making processes.

### 2.3 Step 3: Train, train and train

If the organisation is structured to genuinely prioritise giving patients the most significant voice in their own treatment process, it is then appropriate to commence training. Training will only be effective when the organisation, team structure, and contextual support are properly aligned, and when the nursing mindset is conducive to such efforts (Hakvoort et al. 2021, 2025).

Training should initially target two specific groups: newly graduated nurses who are enthusiastic and open to learning, and nurses entering the profession without preconceived notions (Hakvoort et al. 2022). It is essential to incorporate such training into undergraduate nursing education, as well as to engage these motivated groups early. These individuals can act as change agents, encouraging the rest of the team to follow suit. Organisational support is critical in this process, as young nurses may have limited influence over colleagues. For example, if managers actively endorse the training and encourage its application, its success is far more likely. Therefore, commitment from all organisational levels, particularly management, is imperative before implementing such initiatives (Hakvoort et al. 2025).

Moreover, training programmes should prioritise developing nurses' communication skills, particularly for intercultural interactions, to enable effective engagement with patients from diverse backgrounds (Arends, Thodé, et al. 2022; Bos‐van den Hoek et al. 2021). Many nurses recognise the need for enhanced education in palliative care to facilitate patient‐centred decision‐making (Arends, Thodé, et al. 2022). Effective information‐sharing skills and the ability to manage complex conversations are crucial for supporting SDM (Bos‐van den Hoek et al. 2021). However, limited knowledge often hampers nurses' participation in the decision‐making process (Lenzen et al. 2018; Lewis et al. 2016).

Research demonstrates that decision‐making skills training, particularly through interdisciplinary programmes, enhances communication and leadership, enabling nurses to better fulfil their roles within healthcare teams (Brom et al. 2017; Légaré et al. 2011; Smit et al. 2021). Inter‐professional training, especially for scenarios involving patients with limited life expectancy, improves team coordination and clarifies the roles of both nurses and physicians (Arends, Thodé, et al. 2022). How can these roles be more clearly defined to ensure optimal outcomes?

### 2.4 Step 4: Effective implementation

To effectively implement SDM in daily practice, several strategies are essential for overcoming barriers and promoting adoption, as outlined by Schouten et al. (2022) in their discussion of innovation implementation in healthcare. A critical initial step is identifying and preparing local champions, whose leadership can inspire and guide healthcare teams towards embracing SDM (Smit et al. 2020; Santos et al. 2022). Educational meetings play a vital role in introducing healthcare professionals to the principles and benefits of SDM, fostering their understanding and confidence in collaborative decision‐making (Schouten et al. 2022).

Tailoring SDM approaches to specific patient populations, local contexts, and existing workflows enhances adaptability and ensures seamless integration into clinical routines (Riordan et al. 2022). The provision of comprehensive educational materials, such as toolkits and guides, offers structured frameworks for embedding SDM into practice. Additionally, a trial phase, enabling physicians and nurses to test and refine SDM strategies, helps build trust and competence in its application.

Incorporating transparent, evidence‐based information about SDM processes into existing systems promotes informed and collaborative decision‐making (Riordan et al. 2022). Compatibility with current workflows, while preserving the autonomy of physicians, nurses, patients, and their close relatives, is essential to ensure SDM remains supportive and empowering rather than prescriptive. Emphasising the benefits of SDM—such as improved patient satisfaction, better clinical outcomes, and strengthened provider‐patient relationships—creates a positive impetus for change, motivating healthcare teams to adopt these practices.

These strategies align with the core principles of SDM, advancing patient‐centred care through informed and collaborative decision‐making. However, individual hospitals must adapt and elaborate on each step to suit their unique contexts and develop a detailed implementation plan (Waddell et al. 2021).

By systematically addressing these steps, healthcare organisations can foster an environment that fully supports patient‐centred decision‐making while empowering nurses to play a pivotal role in the process.

### 2.5 Sustainable integration throughout all steps

For successfully going throughout all steps, it is important to note that sustainability should not be considered a distinct step of “sustainable integration” but rather an integral component embedded throughout every stage of the plan presented. From the initial discussions to all phases of development and execution, sustainability must be woven into the process. Collaboration at all levels is essential to evaluate and plan how the intervention will remain viable over time. Building sufficient support for the intervention is equally crucial, alongside a realistic assessment of its feasibility in practice. This avoids creating an idealised plan that cannot be implemented due to constraints such as limited time, inadequate staffing, or insufficient resources. Transparency in every phase is imperative to determine what is achievable and to address potential challenges proactively.

Khalil and Kynoch (2021) propose the Triple C model—comprising Consultation, Collaboration, and Consolidation—which offers a structured framework to integrate sustainability into healthcare interventions. The model emphasises early and ongoing stakeholder engagement, clear communication, and adaptability to local contexts. Gesell et al. (2021) similarly underscore the need to adapt interventions to local settings, actively involve stakeholders, and maintain flexibility in execution. While blueprints for implementation can provide initial guidance, SDM is highly context‐specific, varying significantly across continents, countries, and regions, and evolving over time.

Given the complexity of SDM and the involvement of multiple stakeholders at various stages, continuous evaluation and adaptation are essential for its effective implementation. Finally, monitoring the intervention is crucial to ensuring long‐term sustainability. Khalil and Kynoch (2021) advocate for the use of business intelligence tools tailored to the specific context, enabling the monitoring and refinement of implementation at all levels. These measures ensure genuinely patient‐centred care and support the durability of interventions over time. If all steps are undertaken with a focus on sustainability, the likelihood of achieving genuine SDM may increase significantly.

## 3 Conclusion

A clear plan to realise shared decision‐making has now been outlined, specifically addressing treatment decisions in end‐of‐life care while highlighting its broader applicability across diverse care settings. Although ambitious, this vision is essential for advancing patient‐centred care, and the time has come to translate it into concrete, actionable strategies.

Consider a scenario where a nurse highlights to a physician that not all treatment options have been presented to the patient. In such cases, the absence of explicit policy directives may lead the physician to believe that presenting the most suitable option, thoroughly explained, is in the patient's best interest. However, if the nurse communicates this concern in a blunt and condescending manner, collaboration breaks down. Even if the nurse's point is valid, the counterproductive delivery undermines progress. The physician, constrained by limited time and inadequate organisational support, might justify narrowing the discussion, citing the lack of institutional mechanisms to facilitate these crucial conversations.

When team members demonstrate commitment, clarity, and the necessary skills, these dynamics can change. For instance, the nurse might adopt a more constructive approach by saying, “I know you want what is best for the patient, but we agreed to present all options, and this hasn't been done yet. While you understand what is best, the patient must make the final decision in line with our agreement.” This language promotes collaboration and mutual respect, aligning the team towards shared goals. Such constructive communication, however, requires careful coordination, as outlined in the plan. Success hinges on clear role definitions, institutional commitment, skill development for all stakeholders, and seamless communication. Training is pivotal to ensuring these elements coalesce effectively.

While the plan represents an ideal, we acknowledge that full implementation is neither immediate nor straightforward. It requires time and adaptation. Nonetheless, we challenge hospitals to customise this blueprint to their unique contexts, recognising the variability across countries, institutions, departments, and individuals. To this end, we propose universal components that hospitals can integrate step by step into detailed action plans, starting without delay.

Ultimately, our collective purpose is to achieve optimal outcomes for patients. This shared goal unites physicians, nurses, patients, managers, board members, and other stakeholders in prioritising what is best for the patient. While we continue to navigate the constraints of current systems, it is time to move beyond treating ideal support structures as aspirational. We must turn these principles into practical actions. The plan is ambitious, but it provides a clear path forward. Let us seize this opportunity and work together to make it a reality.

## Author Contributions

Made substantial contributions to conception and design, or acquisition of data, or analysis and interpretation of data; M.T., J.D., P.W.B.N. Involved in drafting the manuscript or revising it critically for important intellectual content; M.T., J.D., P.W.B.N. Given final approval of the version to be published. Each author should have participated sufficiently in the work to take public responsibility for appropriate portions of the content; M.T., J.D., P.W.B.N. Agreed to be accountable for all aspects of the work in ensuring that questions related to the accuracy or integrity of any part of the work are appropriately investigated and resolved: M.T., J.D., P.W.B.N.

## Conflicts of Interest

The authors declare no conflicts of interest.

## Data Availability

The data used are available from the corresponding author upon reasonable request.
